# Picosecond laser-engineered osteon-inspired concentric micropatterns on titanium implants regulate cellular behaviour to facilitate osseointegration

**DOI:** 10.1016/j.mtbio.2025.102704

**Published:** 2025-12-18

**Authors:** Kendrick Hii Ru Yie, Yingyue Sun, Xinhua Gu, Rui Chen, Zhucheng Liu, Qihong Zhang, Lifeng Xiong, Bilal A. Al-Shaaobi, Ahmed S. Mahany, Mingliang Yu, Zhennan Deng, Jinsong Liu, Peng Gao, Lei Lu, Lihua Xu

**Affiliations:** a School and Hospital of Stomatology, Wenzhou Medical University, Wenzhou, 325027, Zhejiang, China; b Zhejiang Glister Photonics, Ltd., Zhuji, 311800, China; c Zhejiang Trusyou Medical Instruments Co., Ltd., 325000, China; d Department of General Medicine, First Affiliated Hospital, Wenzhou Medical University, Wenzhou, 325000, China

**Keywords:** Titanium, Dental implant, Micropatterns, Picosecond laser, Biomimetic

## Abstract

Achieving successful osseointegration in dental implants remains challenging due to biological and biomechanical complications. Inspired by the architecture of osteons, the fundamental structural units of cortical bone, this study employs picosecond-ultraviolet laser (PSL-UV) technology to create biomimetic, osteon-like concentric micropatterns with varying groove widths (20 μm, 40 μm, 60 μm, and 80 μm) on titanium (Ti) surfaces. These patterns aim to regulate cellular behavior and enhance osseointegration. *In vitro* studies demonstrated that groove width critically influenced cellular responses: 20 μm and 80 μm patterns significantly enhanced osteoblast activity while simultaneously regulating or suppressing osteoclast and fibroblastic activity. Gene expression and omics analyses further supported these findings, highlighting the role of micropatterns in modulating cellular differentiation and function. *In vivo* experiments confirmed substantial new bone formation on implants with 20 μm and 80 μm groove widths, underscoring their osteogenic potential. Moreover, the precision and scalability of PSL-UV technology offer a clinically viable solution for improving implant success rates. By tailoring implant surfaces to modulate cellular responses, this strategy enables personalized implant designs, enhancing long-term implant longevity in diverse patient populations. This study provides a promising pathway for advancing dental implantology through biomimetic surface engineering.

## Highlights

• Picosecond laser-fabricated osteon-like micropatterns boost osseointegration.
• 20/80 μm grooves boost osteogenesis and downregulates osteoclast/fibroblast activity.
• Omics-validated multi-pathways activation of enhanced osteogenic responses.
• *In vivo* validation shows augmented bone formation around micropatterned implants.
• Laser technology enables clinical translation of topographically optimized implants.

## Graphical abstract

Schematic diagram illustrating the different types of osteon-like concentric micro patterns and their effects on the different cells (osteoblasts, osteoclasts, and human gingival fibroblasts), and their capacity in augmenting new bone formation for improved osseointegration.

**Figure:**
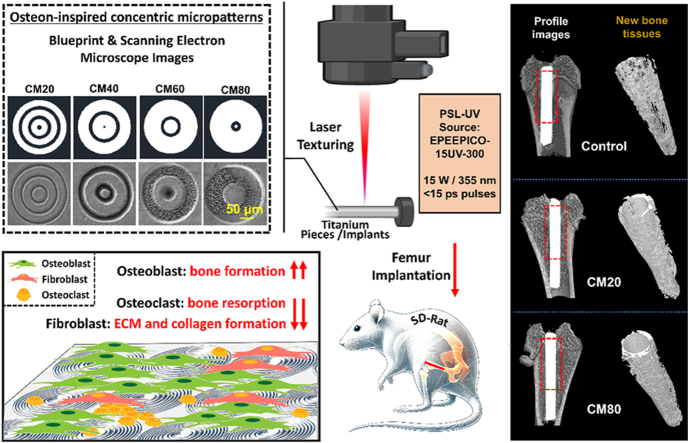

## 1 Introduction

Osseointegration is a fundamental process that ensures the stability and longevity of implants by fostering a direct and functional interface between the implant material and surrounding bone tissue [1,2]. Similar to fracture healing, this process initiates a cascade of biological events, including immune responses, angiogenesis, and recruitment of progenitor cells. A defining feature of osseointegration is the differentiation of these progenitor cells into osteoblasts, which are responsible for synthesizing bone matrix and promoting mineralization [2,3]. Titanium (Ti) and its alloys have become the material of choice for dental and orthopedic implants due to their exceptional strength-to-weight ratio, low density, and corrosion resistance[[4], [5], [6]]. However, achieving successful osseointegration remains a challenge, particularly in the presence of complicating factors such as osteoporosis, diabetes, or bacterial infections, which can disrupt bone remodelling [[7], [8], [9], [10], [11]]. Key cellular players such as osteoclasts and fibroblasts can also undermine implant integration. Excessive osteoclast activity leads to localized bone resorption, destabilizing the implant, while fibroblasts may over-proliferate and form fibrous tissue encapsulating the implant, preventing direct bone-to-implant contact [12,13]. These challenges highlight the need for advanced implant surface modifications strategies that actively guide cellular behavior to optimize implant integration [14,15].

Biomimicry, which draws inspiration from natural systems and structures, has emerged as a transformative strategy in addressing these challenges [16]. The osteon, the fundamental structural unit of cortical bone serves as an ideal model for biomimetic implant design [[16], [17], [18]]. Osteons typically have a diameter ranging from 150 to 350 μm and are composed of concentric lamellae arranged around a Haversian canal measuring approximately 20 ± 10 μm in width in young adults [[17], [18], [19], [20], [21]]. Osteoblasts naturally align along these lamellae, depositing bone in an organized and functional manner for maintaining bone health and adapting of mechanical stresses [22,23]. Hence, mimicking the osteon's architecture on implant surfaces could provide the framework to direct cellular responses, encouraging osteoblast adhesion, migration, and alignment [24].

To translate these biomimetic concepts into practical applications, various surface modification methods, such as lithography and chemical etching, have been employed [25,26]. For instance, one study successfully fabricated concentric micropatterns on polycaprolactone using photolithography, which also demonstrated significant inhibitory effect on osteoclastic activity [19]. However, while these techniques have shown promising results, they often also involve exposing implant materials to polymers and other bioactive components [[27], [28], [29]]. This exposure introduces challenges for clinical adoption, including potential adverse reactions, material damage, and complications in commercialization. These limitations underscore the need for alternative approaches that are both more biocompatible and scalable for widespread clinical adoption.

Advances in laser technology, such as the picosecond-ultraviolet laser (PSL-UV) used in this study, provides a more impactful solution for overcoming the limitations of conventional surface modifications [[30], [31], [32], [33]]. By adjusting laser parameters, this approach allows for precise and selective modifications of critical surface properties, including topography, roughness, and wettability, while preserving the internal structure and bulk properties of the material [[33], [34], [35]]. This precision allows for the fabrication of the osteon-inspired concentric micropatterns herein, directly onto Ti surfaces. Additionally, the flexibility of this approach also allows for the introduction of varying groove widths within the micropatterns to investigate the effects of micro-topographical cues on the behavior of osteoblasts, osteoclasts, and fibroblasts [[36], [37], [38]]. This simple yet effective approach does not only enable more suitable biomimetic implant designs but also address the practical challenges in surface modification for enhancing implant integration.

**Scheme 1:**
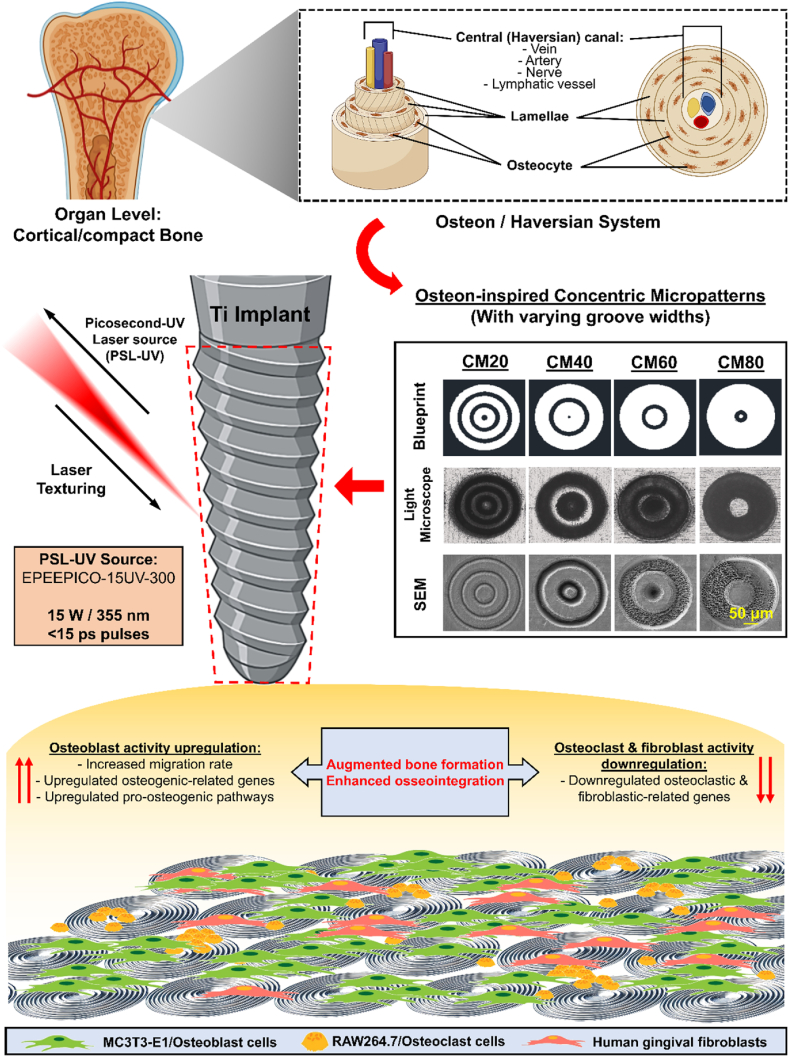
Schematic illustration depicting the biomimetic approach for enhancing implant osseointegration *via* direct picosecond-UV laser-texturing of osteon-inspired concentric micropatterns with varying groove widths on Ti implants. These structures promoted osteoblast activity while regulating/inhibiting osteoclast and fibroblast activity, resulting in augmented bone formation and improved osseointegration.

This study proposes that osteon-inspired concentric micropatterns, produced *via* direct PSL-UV laser-texturing, can improve osseointegration by promoting osteoblast activity while regulating and/or inhibiting osteoclast and fibroblast activity. Through systematic physicochemical, *in vitro* and *in vivo* investigations, the research also explores how varying groove widths influence cellular behavior and bone regeneration, offering valuable insights into the potential of these osteon-inspired concentric micropatterns in advancing implant technology while demonstrating potential for personalized medicine, enabling dental implants tailored to patient needs (Scheme 1).

## 2 Materials and methods

### 2.1 Materials

Ti foils (0.5 mm thickness) were purchased from Baoji Fuxin Non-ferrous Metal Products Co. Ltd. (Xi'an, China). Ti rods (1.2 mm in diameter) were provided by Zhejiang Trusyou Medical Instruments Co., Ltd. (China). 3-(4,5-dimethylthiazol-2-yl)-2,5-diphenyltetrazolium bromide (MTT), phosphate buffered saline (PBS), alizarin red-S solution, 2.5 % glutaraldehyde fixation solution, and tartrate-resistant acid phosphatase (TRAP) stain kit were purchased from Solarbio Co. (Beijing, China). Alkaline phosphatase (ALP), bicinchoninic acid (BCA), cell counting kit-8 (CCK-8), 5-bromo-4-chloro-3-indolyl-phosphate/nitro blue tetrazolium (BICP/NBP) ALP color development kit, TRAP assay kits, 4 % paraformaldehyde fix solution, 4′,6-diamidino-2-phenylindole (DAPI) dye, FITC-phalloidin dye were provided by Beyotime Biotech Co. (Jiangsu, China). Finally, mouse recombinant receptor activator of nuclear factor κ-B ligand (RANKL) and macrophage colony-stimulating factor (m-CSF) were purchased from PeproTech, Inc. (NJ, USA).

### 2.2 Fabrication of osteon-inspired concentric micropattern samples

Ti pieces (10 mm × 10 mm × 0.5 mm) were washed with pure ethanol and deionized (DI) water under ultrasonication to remove any impurities and contaminants. The osteon-inspired concentric micropatterns were first designed in CAD software (CorelDrawX5, Cascade Parent Limited, Canada), where the concentric layout, groove width, wall width, and pattern spacing were defined. These CAD designs were then imported into the Scanworld laser processing software for fine tuning and alignment before laser texturing.

The geometric dimensions of the micropatterns were fixed across all groups except for groove width. Each concentric unit had a diameter of 200 μm, a groove wall width of ∼15 μm, a spacing of 30 μm between adjacent micropatterns, and a groove depth target of ∼20 μm. The groove widths were set at 20, 40, 60, or 80 μm for the CM20, CM40, CM60, and CM80 groups, respectively. These structural parameters formed the basis for laser processing and were later confirmed using SEM and 3D surface profilometry.

The samples were subsequently textured using a 15 W, 355 nm PSL-UV laser (Zhejiang Glister Photonics Co. Ltd., China) with a pulse width of up to 15 ps at a fixed repetition frequency of 300 kHz and a scan speed of 200 mm/s. The laser system was coupled with a × 10 beam expander and a Hurryscan II 14 (Scanlab) galvo scanner equipped with a 110 mm focal-length telecentric F-theta lens. To ensure consistent micropattern design, scan speed (200 mm/s), and frequency (300 kHz) were kept constant. Laser power (30–35 %) and the repetition rate were adjusted to obtain a consistent groove depth of ∼20 μm. The full laser and micropattern parameters are listed in Table S1.

**Fig. 1:**
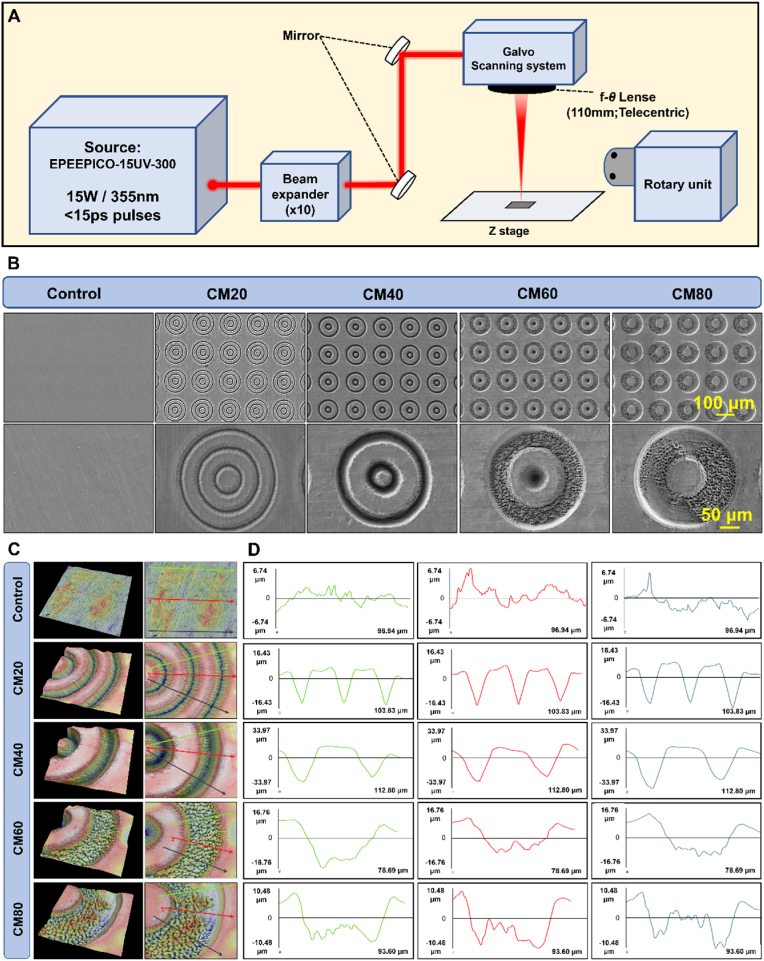
**Fabrication and characterization of the osteon-inspired concentric micropattern samples (CM20, CM40, CM60, CM80). A)** Illustration depicting the PSL-UV setup used in this study; **B)** SEM of the different samples; **C)** 3D surface profiles, and 2D height images; **D)** Cross-sectional profiles of the samples along the lines marked in **C.**

Post-processing, samples were further rinsed in DI water and ethanol via ultrasonication and stored in vacuum-sealed containers. The full laser setup is illustrated in Fig. 1A. It should be noted that non-laser textured polished Ti was selected as the baseline control to ensure that the biological responses observed in this study could be attributed specifically to the engineered concentric micropattern geometry.

### 2.3 Sample characterization

The utilization of scanning electron microscopy (SEM, SU-8010, Hitachi, Japan) and a desktop scanning electron microscope (Phenom Pharos, Thermo Fisher Scientific, U.S) allowed for the observation of topological characterizations pertaining to the different Ti substrates. In order to gain insight into the elemental distribution and the phase structure of the various samples, an energy-dispersive X-ray spectroscope (EDS) integrated desktop scanning electron microscope (Phenom Pharos, Thermo Fisher Scientific, U.S) and an X-ray powder diffraction (XRD, ThermoFisher, USA) was utilized, respectively. Furthermore, the measurement of the water contact angle (WCA) of the samples was facilitated through the employment of a contact angle goniometer (SDC-200S, Sindin, China).

### *2.4* *In vitro* osteogenic activities

#### 2.4.1 MC3T3-E1 cell culture

The murine derived osteoblast precursor cell line, MC3T3-E1 cells, were cultured in a 10 % fetal bovine serum (FBS) and 1 % penicillin/streptomycin supplemented α-MEM medium at 37 °C under 5 % CO_2_ atmosphere. The cells were passaged at around 80 % confluence rate. All samples were disinfected through immersion in 75 % ethanol and ultraviolet (UV) irradiation for 40 min each side.

#### 2.4.2 MC3T3-E1 cell morphology observation

The cells were cultivated on the various samples (Control, CM20, CM40, CM60, and CM80) at a density of 8 × 10^3^ cells/cm^2^ for a duration of 3 days (n = 6 samples/group). Following this, the adherent cells were fixed using a solution of 4 % paraformaldehyde for a period of 1 h and subsequently treated with 0.2 % Triton X-100 (SolarBio, China) in order to create perforations in the cell membrane. FITC-phalloidin and DAPI dyes were utilized to stain the cell cytoskeleton and nucleus respectively, with a staining duration of 1 h for the former and 10 min for the latter. Finally, an inverted fluorescence microscope (IFM, OLYMPUS IX71, Japan) was employed to observe the cells that had been stained.

High-resolution images of cellular morphology were captured and examined through the use of an SEM. Prior to this, the samples were prepared in a similar fashion as mentioned above. In summary, following cell culture and fixation with paraformaldehyde, the adherent cells underwent a dehydration process utilizing a series of graduated ethanol solution (30 %, 50 %, 70 %, 90 %, 100 % v/v) as well as hexamethyldisilazane (HMDS) solution for critical-point drying. Subsequently, in order to obtain SEM images with enhanced contrast and intricate details, the samples were subjected to a platinum particle sputter (EM ACE600, Leica Microsystems, Germany) within a vacuum chamber for a duration of 80 s before observation. To visually indicate the presence of cells on the substrates, a software known as Mountains SEM (Digital Surf, France) was utilized for the colorization of the SEM images.

For quantitative analysis of MC3T3-E1 cell distribution relative to the micropatterns, fluorescence images were analyzed using ImageJ (NIH, U.S.). For each sample, four non-overlapping fields of view were randomly selected at a fixed magnification using the inverted fluorescence microscope. In each field, the number of cells located within the micropatterned (groove) region and on the surrounding smooth Ti surface was manually counted and normalized to the corresponding surface area. The percentage of cells located inside versus outside the micropatterns was then calculated (n = 6 samples/group).

#### 2.4.3 MC3T3-E1 cell viability

The viability of MC3T3-E1 cells was assessed by employing the MTT assay. In brief, the cells were cultured on the different samples at an initial density of 2 × 10^4^ cells/cm^2^ for 3 and 7 days (n = 6 samples/group for both time points). Following each time point, a solution of 0.9 mL of fresh α-MEM culture medium supplemented with 0.1 mL of MTT (5 mg/mL) was added to each well, and incubated for an additional 4 h at 37 °C. Subsequently, the MTT-supplemented culture medium was removed and replaced with 0.5 mL of dimethyl sulfoxide (DMSO) to dissolve the formazan crystals that had formed. Finally, the optical density of the resulting solution was measured at 490 nm using a spectrophotometric microplate reader (Bio-Rad 680, Bio-Rad Laboratories Inc., U.S).

#### 2.4.4 MC3T3-E1 cell adhesion and migration

MC3T3-E1 cells were seeded onto the substrates at an initial density of 8 × 10^3^ cells/cm^2^ for 2 h (n = 6 samples/group). A similar approach was employed for the fluorescence observation of the MC3T3-E1 cell adhesion. Firstly, 0.2 % Triton X-100 was used to perforate the cell wall to facilitate better staining. Next, the cells were dyed using DAPI dye for 10 min and observed under an inverted fluorescence microscope. The ImageJ software (NIH, U.S) was used to quantify the of the adherent cell count.

Ti samples (10 mm × 20 mm × 0.5 mm) were bent into an L-shape, with one face consisting of the smooth untreated Ti surface and the adjacent face containing the micropatterns. MC3T3-E1 cells were seeded onto the smooth surface at a density of 8 × 10^3^ cells/cm^2^ and allowed to adhere for 6 h (n = 6 samples/group). The samples were then rotated by 90°, placing the micropatterned face on the bottom of the well to allow cells to migrate downward from the seeded region toward the micropatterned area. At 12 h and 24 h (performed as independent endpoints), samples were rinsed with PBS, fixed with 4 % paraformaldehyde for 1 h, permeabilized with 0.2 % Triton X-100, and stained with FITC-phalloidin. An inverted fluorescence microscope at a fixed magnification was used to determine the migration of the cells. The imaging began at the boundary where the smooth surface ended and the micropatterns began, which served as the reference point for all measurements. Multiple images of the migration were captured along the horizontal axis and stitched together using the Photoshop 2020 software (Adobe, U.S) to reconstruct the full migration path. Migration distance was quantified in ImageJ (NIH, U.S.) by measuring the displacement of the leading cell front from the reference boundary. For each sample, a minimum of two non-overlapping regions were analyzed. This procedure was adapted from previously described method [39].

#### 2.4.5 ALP staining and activity

To investigate the capacity of MC3T3-E1 cells for osteogenic differentiation, the determination of ALP activity was conducted. Firstly, the cells were seeded onto various substrates at a density of 2 × 10^4^ cells/cm^2^ for a period of 3 and 7 days (n = 6 samples/group for each time point). Following each time point, 1 % Triton X-100 was introduced for the purpose of cell lysis. Subsequently, the protein concentration and ALP activity were measured at 562 nm and 520 nm, respectively, utilizing BCA protein and ALP assay kits with the aid of a spectrophotometric microplate reader (Bio-Rad 680, Bio-Rad Laboratories Inc., U.S). ALP activity was normalized to the corresponding total protein content for each sample.

To visualize the staining of ALP, at each pre-determined time point, cells were immobilized using a 4 % paraformaldehyde solution for a duration of 1 h. Subsequently, the cells were rinsed with PBS and subjected to staining according to the instructions outlined in the BCIP/NBT ALP staining kit, under dim lighting conditions, for a period of 40 min. Ultimately, the samples underwent further washing to remove any excess stain, after which the stained specimens were captured utilizing a stereoscopic microscope (NIKON, Japan).

#### 2.4.6 Mineralization quantification and staining

The utilization of alizarin red-S dye, a well-known dye utilized for the purpose of staining calcium-containing osteocytes, was employed in order to assess and analyze the mineralization matrix of MC3T3-E1 cells. In brief, the cells were seeded onto various samples at a density of 2 × 10^4^ cells/cm^2^ for 14 and 21 days (n = 6 samples/group for each time point). After each time point, the samples were thoroughly washed with PBS and 4 % paraformaldehyde was used for cell immobilization for a duration of 1 h. Next, 0.3 mL of alizarin red-S dye was added to stain the samples for 30 m. The samples were then washed again with DI water before observation under a stereomicroscope. Consequently, the calcium nodules that had been stained were dissolved in a solution of 10 % cetylpyridinium chloride for a duration of 120 min, and then the absorbance was measured at a wavelength of 540 nm using a spectrophotometric microplate reader (Bio-Rad 680, Bio-Rad Laboratories Inc., U.S).

#### 2.4.7 Osteogenic gene expression

MC3T3-E1 cells (2 × 10^4^ cells/cm^2^) were seeded onto the different samples (n = 6 samples/group). After 7 and 14 days, the total RNA was isolated using TRIzol and an RNA extraction kit (Tiangen, China), followed by a subsequent total RNA concentration evaluation using NanoDrop 2000 Spectrophotometer (Thermo Fisher Scientific, USA). Next, the PrimeScript™ RT reagent kit (TaKaRa, Japan) was used to reverse transcribe RNA into cDNA, with 1 μg of total RNA used per reaction according to the manufacturer's protocol. Finally, the SYBR Premix ExTMTaq II kit (TaKaRa, Japan) was utilized to verify the expressions of the various target genes (runt-related transcription factor 2 (*Runx2*), Alkaline phosphatase (*Alp*), collagen I (*Col1*), osteocalcin (*Ocn (Bglap)*), and osteoprotegerin (*Opg*)). The housekeeping gene, glyceraldehyde 3-phosphate dehydrogenase (*Gapdh*) was used to normalize the relative expressions of the aforementioned genes. The list of primers is shown in Table S2.

#### 2.4.8 Transcriptomic analyses

For RNA sequencing (RNA-seq) analyses, 3 groups were chosen (Control, CM20, and CM80). In brief, MC3T3-E1 cells (3 × 10^4^ cells/cm^2^) were seeded onto the samples (4 cm × 4 cm; n = 6 samples/group). After 7 days, the cells were collected, stored in TRIzol solution, frozen at −80 °C, and sent for RNA-seq analyses (Majorbio Biotech, Shanghai, China). Differentially Expressed Genes (DEGs) were set at |log2FC| > 1.0 and *p* < 0.05.

### *2.5* *In vitro* osteoclastic activities

#### 2.5.1 Osteoclasts differentiation from RAW 264.7 cells

The murine derived macrophage/monocyte cell line, RAW264.7 cells, were cultured in a 10 % FBS, 1 % penicillin/streptomycin supplemented high-glucose Dulbecco's modified eagle medium (DMEM) at 37 °C under 5 % CO_2_ atmosphere. The cells were passaged at around 80 % confluence rate. RANKL (50 ng/mL) and macrophage colony stimulating factor (m-CSF, 20 ng/mL) were only added into the culture media for osteoclastic differentiation studies of RAW264.7 cells. All samples were disinfected through immersion in 75 % ethanol and UV irradiation for 40 min each side.

#### 2.5.2 Osteoclast morphology observation

RAW264.7 cells (3 × 10^4^ cells/cm^2^) were seeded onto the samples and cultured for 4 days under the osteoclast-differentiation conditions described in section 2.5.1 (n = 6 samples/group). The fluorescent staining, SEM imaging sample preparation procedures, and cell distribution quantification of RAW264.7 cells were identical to that of osteoblast cells, with reference to section 2.4.2.

#### 2.5.3 TRAP staining and activity

RAW264.7 cells (3 × 10^4^ cells/cm^2^) were seeded onto the samples and cultured for 4 days following the osteoclast-induction protocol described in section 2.5.1 (n = 6 samples/group). Parallel cultures were used for TRAP staining and TRAP activity analysis.

For TRAP staining, the samples were fixed in TRAP fixative solution for 3 min at 4 °C, then were washed using DI water and left to dry slightly at room temperature. Subsequently, the TRAP incubation solution was added into the samples and was incubated at 37 °C for 60 min. The solution was then discarded and the samples were rinsed again with DI water. Finally, hematoxylin dye was applied for 3 min and the stained samples were examined under a stereomicroscope.

For TRAP activity analysis, parallel cultures were processed using a TRAP assay kit (Beyotime, China). In brief, cells were permeabilized with 1 % Triton X-100 for 30 min at room temperature, after which 40 μL chromogenic substrate and 5 μL p-nitrophenyl phosphate (0.5 mM) were added and incubated at 37 °C for 5 min. Finally, 160 μL of stop buffer was added to terminate the reaction and the data were quantified using a microplate reader (BioRad 680, USA) at 405 nm wavelength.

#### 2.5.4 Osteoclastic gene expression

RAW264.7 cells (3 × 10^4^ cells/cm^2^) were seeded onto the samples and cultured for 4 days following the same osteoclast-induction procedure described in section 2.5.1 (n = 6 samples/group). After 4 days, the total RNA was then extracted and the expressions of the various target genes (receptor activator of nuclear factor κ-B (*Rank*), tartrate phosphatase (*Trap (Acp5)*), and cathepsin K (*Ctsk*)) were detected and normalized with the *Gadph* gene following an identical experimental protocol with reference to section 2.4.7. The list of primers is shown in Table S2.

### *2.6* *In vitro* fibroblastic activities

#### 2.6.1 Human gingival fibroblasts (HGFs) culture

HGFs were cultured in a 10 % FBS, 1 % penicillin/streptomycin, 4 mM L-Glutamine, 110 mg/L sodium pyruvate, and fibroblast growth factor supplemented high-glucose DMEM at 37 °C under 5 % CO_2_ atmosphere. The cells were passaged at around 80 % confluence rate. All samples were disinfected through immersion in 75 % ethanol and UV irradiation for 40 min each side prior to usage.

#### 2.6.2 HGF morphology observation

The cells were cultured onto the different samples at an initial density of 1 × 10^4^ cells/cm^2^ for 3 days (n = 6 samples/group). The fluorescent staining, SEM imaging sample preparation procedures, and cell distribution quantification of HGF cells were identical to section 2.4.2, 2.5.2.

#### 2.6.3 HGF viability

CCK-8 was used to determine the viability of the HGFs. HGFs at an initial density of (2 × 10^4^ cells/cm^2^) were cultured on the different samples in a 24-well plate for 4 and 7 days (n = 6 samples/group for each time point). After each time point, the medium was discarded, and then a 400 μL of culture medium supplemented with 10 % CCK-8 solution was added into each well and was further incubated for 2 h. Next, 200 μL of the CCK-8/medium solution was transferred to a 96-well plate and the absorbance value was determined using the spectrophotometric microplate reader at 450 nm.

#### 2.6.4 Fibroblastic gene expression

HGFs (5 × 10^4^ cells/cm^2^) were cultured on various samples for 7 days (n = 6 samples/group). An identical method to the osteoblastic and osteoclastic gene expression was employed (with reference to section 2.4.7, 2.5.4, respectively). The expressions of the various target genes (type 1, alpha 1 collagen (*COL1A1*), integrin alpha 3 (*ITGA3*), integrin beta 1 (*ITGB1*), fibronectin 1 (*FN1*), and vinculin (*VCL*)) were detected and normalized with the *GAPDH* gene. The list of primers is shown in Table S2.

### *2.7* *In vivo* biocompatibility

#### 2.7.1 Implants preparation

Based on the collated *in vitro* results, 3 groups (Control, CM20, and CM80) were selected for the *in vivo* study. In brief, Ti rods (1.2 mm in diameter, 12 mm in length) were cleaned through ultrasonication using DI water and pure ethanol prior to laser texturing. The designs of the osteon-inspired concentric micropatterns as well as the laser parameters were kept identical to the *in vitro* samples with reference to section 2.2 and Table S1. The Ti rods were clamped onto a high-precision, motorized rotary stage. It should be noted that the Ti rods were intentionally cut 12 mm long to allow for clamping. Initially, a single line of micropatterns were engraved onto the full length of the Ti rod (∼10 mm) followed by a rotation of the sample then the engraving of the subsequent line of micropatterns. This process will continue until the entirety of the substrate is adorned with the micropatterns. Next, the excess length (∼2 mm) of the samples was cut and discarded. The samples were again washed and cleaned through ultrasonication to remove any impurities, debris, and contaminants. Finally, all samples were disinfected and sterilized under submersion in 75 % ethanol and UV exposure, dried, and stored in a vacuum container until needed.

#### 2.7.2 Surgical protocol and procedure

The *in vivo* experiments were conducted in compliance with the regulations and guidelines established by the Animal Ethics Committee of Wenzhou Medical University (accreditation number: xmsq2022-0845). A total of 15 Specific-pathogen-free (SPF) graded Sprague-Dawley (SD) rats (female, 8–9 weeks old, 200–300 g in weight) were purchased from Charles River Laboratories (U.S). The rats were housed in a sterilized and controlled environment (25.5 ± 0.5 °C, 50–70 % relative humidity) and were given pathogen-free rat chow and water *ad libitum*. The rats were then randomly divided into groups of three for the distal femoral epiphysis implantation procedure. Each rat was implanted with two different samples and the groupings are as follows: 1) Control/CM20; 2) CM20/CM80; 3) CM80/Control.

After one week of acclimatization, the rats were anesthetized through intraperitoneal administration of pentobarbital sodium (30 mg/kg) followed by shaving and disinfection of the surgical sites using iodine solution. A single incision was made on both patellar region of both legs to expose the femoral epiphysis. A surgical drill fitted with a fissure bur was used to create a cylindrical cavity (10 mm depth, 1.2 mm in diameter) for implant placement (Fig. 7A). Normal saline was used for irrigation to dissipate heat generated from the drilling process. Interrupted sutures were then employed to close the incision. Intramuscular injection of ampicillin antibiotic (10–15 mg/kg) was administered to the rats in the first three days post-surgery. It should be highlighted that all surgical instruments were sterilized and disinfected prior to usage.

#### 2.7.3 Micro-CT evaluation

After a duration of one month following the implantation, the rats were humanely killed and the femurs were gathered. The bone samples were then preserved at a temperature of 4 °C in a solution of 4 % paraformaldehyde. In order to visualize and assess the localized osteogenic capacity of the various implants, the process of new bone regeneration was observed using a micro-CT scanner (Skyscan1176, Bruker, Germany). Each bone sample was individually affixed to the sample tray and subsequently subjected to a 360° rotating X-ray beam (80 kV, 300uA), scanning at an average rate of 2 frames per 0.5° rotation step. The obtained data was then analyzed and quantified using the software Data Viewer, CTVox, CTAn, and CTVol (Bruker, Germany).

#### 2.7.4 Histological analyses

Following micro-CT analysis, the samples underwent de-calcification using ethylenediaminetetraacetic acid (EDTA, 12 %) for a duration of 21 days. Subsequent to the de-calcification process, the various implants were cautiously extracted to safeguard the newly developed bone surrounding them. Following this, all bone specimens were dehydrated through a series of ethanol solutions of varying concentrations (10 %, 30 %, 50 %, 70 %, 90 %, and 100 %, v/v) before undergoing paraffin fixation. Lastly, histological examinations were conducted using hematoxylin and eosin (HE) as well as Masson's trichrome (MT) staining techniques.

### 2.8 Statistical analyses

To ensure the reliability and reproducibility of our findings, each *in vitro* experiment was carried out using n = 6 samples per group per time point and was independently repeated at least three times. Data are presented as mean ± standard deviation (SD). To assess and measure the significance of the obtained data, the one-way analysis of variance (ANOVA) method was employed, followed by Tukey's multiple comparison test. All analyses were performed at a 95 % confidence level using GraphPad Prism 7.0 (San Diego, CA, USA).

## 3 Results and discussion

### 3.1 Surface characterizations

In this study, we fabricated micropatterns that mimic the osteon, focusing on their concentric structure. The designed dimensions (Table S1) closely align with natural osteons with CM20 group (groove width of 20 μm) bearing the most resemblance. This study explores on how varying groove widths affect the behavior of osteoblasts, osteoclasts, and fibroblasts.

SEM images (Fig. 1B) confirmed that the texturing of the micropatterns were successful and were consistent with the blueprint of the micropatterns. All groups (CM20, CM40, CM60, and CM80) exhibited their intended concentricity, diameter (∼200 μm), and groove width (20, 40, 60, and 80 μm, respectively). The measured WCA for both the CM20 (55.1 ± 6.8°) and CM40 (57.2 ± 11.9°) groups were lower when compared to the control (77.2 ± 6.3°) group indicating that both CM20 and CM40 were more hydrophilic (Fig. S1). In contrast, the CM60 (85.4 ± 4.4°), and CM80 (83.3 ± 3.9°) were more slightly more hydrophobic than the control with a WCA measurement of 85.4 ± 4.4° and 83.3 ± 3.9°, respectively. There was, however, no significant differences between the micropattern groups and the control. These differences, although small, may be attributed to the dimensions and the total surface area of each micropattern design. It should also be highlighted that the increase in wettability of these surfaces may be beneficial to initial adhesion and anchorage of the cells.

To further confirm the parameters and dimensions of these micropatterns designs, specifically groove depth, 3D reconstructed images were made using the Phenom Pharos software. As illustrated in Fig. 1C, the 3D surface profiles show the expected rings of groove indentations on the samples. Furthermore, three horizontal lines (green (G), red (R), blue (B)) were drawn outwards for each sample beginning from the centre of the micropattern to analyze the 2D cross-sectional surface height profiles (Fig. 1D). By calculating the average vertical height (from the highest peak to lowest valley) of multiple points along a single line, the Rz value for the various groups were determined as the groove depth: 1) Control (G: 3.27 μm; R: 4.60 μm; B: 4.36 μm), 2) CM20 (G: 16.54 μm; R: 16.90 μm; B: 19.46 μm), 3) CM40 (G: 25.75 μm; R: 25.76 μm; B: 27.53 μm), 4) CM60 (G: 10.46 μm; R: 7.90 μm; B: 9.07 μm), 5) CM80 (G: 7.39 μm; R: 9.38 μm; B: 8.80 μm). It is also important to highlight that the resultant grooves of the micropatterns were slightly tapered. The original intent was to create a groove with right angle floors, however due to inherent limitation of laser technology, this could not be realized. The observed structural anomalies of the micropatterns, specifically the bead-like and bulbous malformations on the floor of the grooves of the CM40, CM60, and CM80 groups, are hypothesized to be attributable to initial errors in laser or design parameters, as well as excessive heating from the PSL-UV technique. The average groove depth of approximately 20 μm, excluding defects, was corroborated through measurements, and the presence of structural anomalies did not hinder subsequent analyses.

After laser-processing, XRD was employed to determine the crystalline structure of the different groups. As shown in Fig. S2, the observable peaks of the experimental groups were identical to that of the control group suggesting that the laser-marking process has little to no effect in causing Ti phase transformations such as characteristic peaks of anatase or rutile phases which have been reported to have osteogenic and anti-microbial effects [[40], [41], [42]]. This confirmed that observed *in vitro* results were solely due to the micropatterns.

Furthermore, to measure the elemental compositions of the samples, EDS analysis was carried out. There were only Ti, oxygen (O), and carbon (C) elements present on the samples according to the presence of their representative peaks in Fig. S3. Shown in Table S3, no significant changes could be observed in the weight percentage (Wt%) and atomic percentage (At%) of C among the groups. However, we were able to confirm that the Ti samples have undergone an oxidation process as a consequence to the laser-texturing process. The Wt % and especially the At % of Ti in the control group (89.98 % and 74.90 %, respectively) has significantly decreased (10–20 %) when compared to the micropatterned groups. In contrast, a significant increase of O (10–20 %) was also observed in all of the micropatterned groups.

### *3.2* *In vitro* osteogenic evaluation

#### 3.2.1 Effect of the osteon-inspired concentric micropatterns on the morphology and distribution of MC3T3-E1 cells

**Fig. 2:**
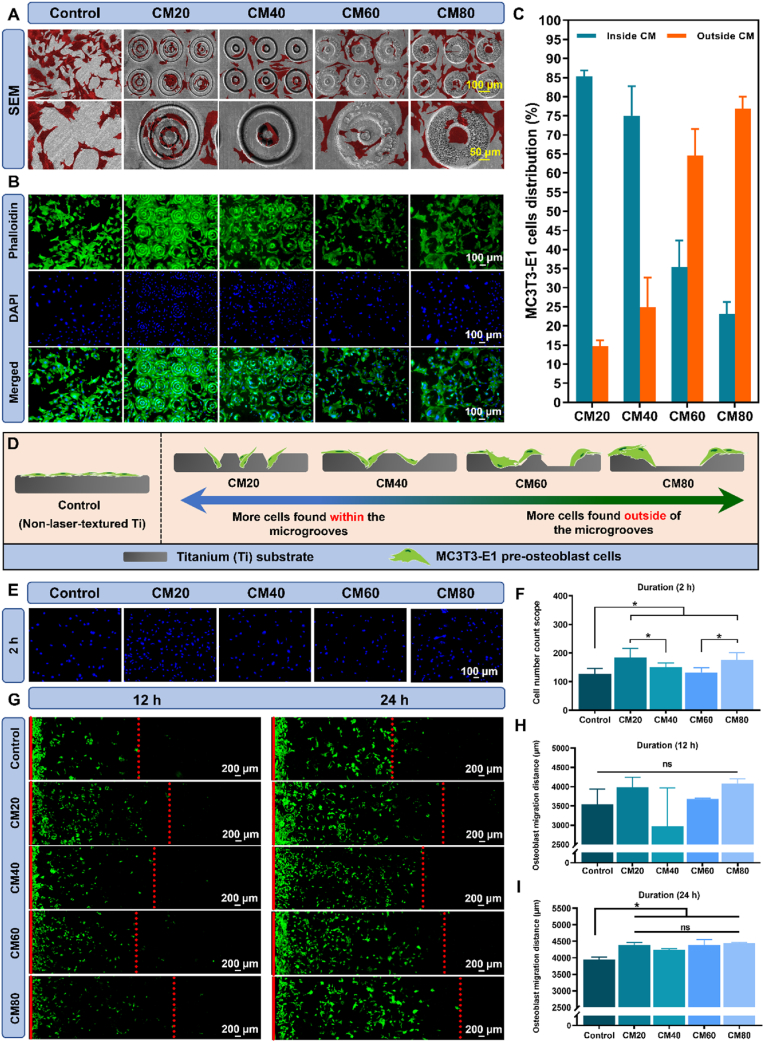
**MC3T3-E1 pre-osteoblast cells behavioural characterization on the different osteon-inspired concentric micropatterns. A)** SEM images of MC3T3-E1 cells on the different samples (cells are highlighted in red colour); **B)** Fluorescence staining of MC3T3-E1 cells using FITC-phalloidin and DAPI staining on the different samples; **C)** Average distribution of MC3T3-E1 cells on the samples in percentage; **D).** Schematic diagram depicting the distribution, preference, and selectivity of MC3T3-E1 cells of the different osteon-inspired concentric micropatterns. **E)** Visualization of MC3T3-E1 cell adhesion on the different samples after 2 h by DAPI staining; **F)** Cell count number of adhered MC3T3-E1 cells on the different samples after 2 h; **G)** Fluorescence staining using FITC-phalloidin on the migration of MC3T3-E1 cells on the different samples after 12 h and 24 h; **H & I)** Quantified migration distance of MC3T3-E1 cells after 12 h and 24 h, respectively. Multiple images were taken and stitched together using Adobe photoshop. **∗***p* < 0.05.

The SEM images (Fig. 2A) of the CM20 group demonstrated that the MC3T3-E1 cells were primarily concentrated within the grooves of the micropatterns, exhibiting an elongated morphology. Furthermore, these cells conformed to the curvature of the grooves. This phenomenon is referred to as contact guidance which is defined as the ability of cells to orient themselves and elongate based on the topographical features of their micro-environment [43,44].

In contrast, we can observe a minimal number of cells within the micropatterns in the CM80 group, as the majority of cells were situated on the smooth Ti surface outside of the micropatterns. Notably, these cells possessed a wider cell area, with their lamellipodia extending and adapting to the edges of the micropatterns. As for the CM40 and CM60 groups, a combination of these two distribution patterns can be seen. However, the cells in these groups tended to favour the narrower groove width of the CM40 group. This observation was further confirmed through the FITC-phalloidin/DAPI staining images (Fig. 2B). The cells were clearly and distinctly situated within the grooves of the CM20 group, and as the width of the groove increases, the cells position themselves outside of the micropatterns. It is worth emphasizing that the extent of cell spreading on the CM80 group is comparable, if not greater, than the spreading of cells on the control group. The quantified analysis of these observations is shown in Fig. 2C. The schematic diagram (Fig. 2D) illustrates the distribution, preference, and selectivity of MC3T3-E1 cells across various micropatterned surfaces.

#### 3.2.2 Effect of the osteon-inspired concentric micropatterns on the adhesion and migration of MC3T3-E1 cells

Fig. 2E exhibits the visual representation of the DAPI staining images, which serve to depict and elucidate the initial adhesion process of the MC3T3-E1 cells onto the samples after a duration of 2 h. Upon careful observation and analysis, it becomes apparent that both the CM20 and CM80 groups exhibit a substantially higher number of cells that have successfully adhered to the substrate. The quantified analysis (Fig. 2F) revealed that both the CM20 and CM80 groups exhibited significantly higher number of adhered cells than other groups, with a count of 184 ± 32 cells and 175 ± 26 cells, respectively. Conversely, no statistically significant differences in the number of adhered cells were observed between the control group, CM40 group, and CM60 group.

The migration assay, which can be seen in Fig. 2G–I, also demonstrated a similar trend as mentioned above. The CM20 and CM80 groups were found to display the most extensive migration distances, indicating that they possessed the greatest augmented migration rate, which was measured at approximately 3987.1 ± 257.5 μm and 4076.3 ± 124.7 μm, respectively, following a 12-h incubation period. However, it is important to note that there were no statistically significant differences observed among the various groups. Conversely, after 24 h, all of the micropatterned groups displayed a significant increase in cellular migration distance, surpassing the distance of 4200 μm, whereas the control group reached its peak at around 3948.4 ± 68.3 μm.

The enhanced adhesion and migration mentioned above groups may be attributed primarily to the guidance cues provided by their microgroove architecture. However, previous studies have reported that ultrafast laser–induced surface oxidation could further promote early cell adhesion and migration on Ti substrates [45,46]. Consistent with these findings, our EDS analysis mentioned in section 3.1, Fig. S3, and Table S3, revealed mild surface oxidation on the PSL-UV–textured samples. This suggests that, in addition to the geometric effects of the concentric micropatterns, the oxidized surface layer may also contribute to the improved adhesion and migration behaviors observed in the concentric micropattern groups.

#### 3.2.3 Cytocompatibility and osteogenic differentiation of MC3T3-E1 cells on the different osteon-inspired concentric micropatterns

**Fig. 3:**
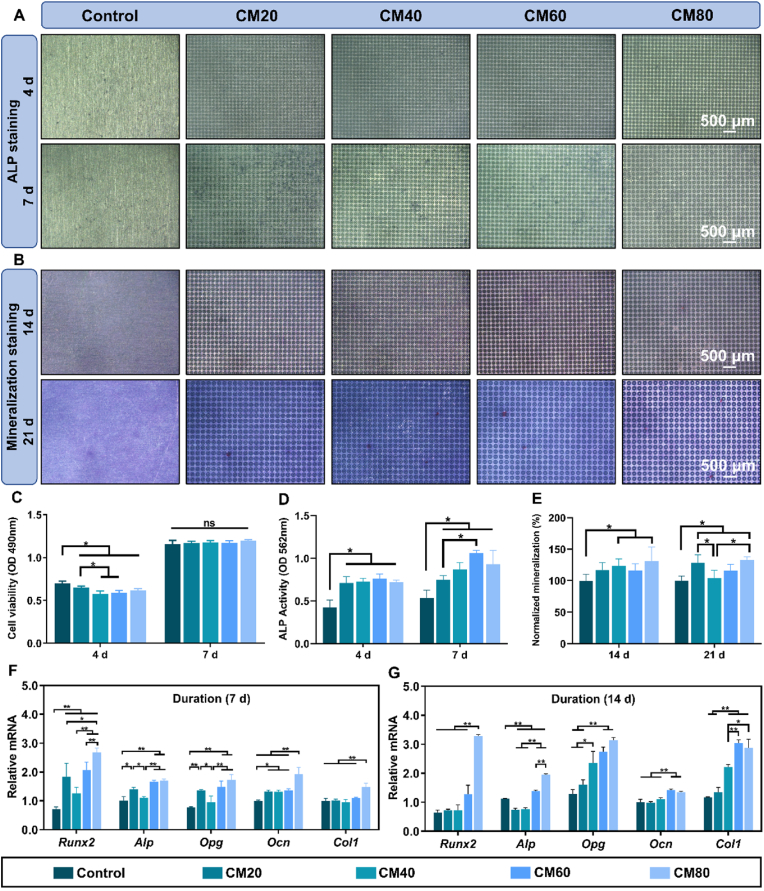
**Osteogenic activity of MC3T3-E1 pre-osteoblast cells on the different osteon-inspired concentric micropattern samples. A)** ALP staining of the different samples at 4 and 7 days (purple-colored stains); **B)** Alizarin red-S staining of the different samples to visualize mineralization at 14 and 21 days (red-colored stains and nodules); Quantified analyses of **C)** cell viability, **D)** ALP activity at 4 and 7 days, and **E)** percentage mineralization activity at 14 and 21 days; **F & G)** Relative osteogenic gene expressions (*Runx2*, *Alp*, *Opg*, *Ocn*, and *Col1*) measured at 7 and 14 days, respectively. ∗*p* < 0.05, ∗∗*p* < 0.01.

As depicted in Fig. 3C, on the fourth day, the viability of cells in the control group was markedly superior when compared to the experimental groups. Nevertheless, by the seventh day, all micropatterned groups exhibited viability levels comparable to that of the control group. These results imply that, notwithstanding the laser-marking procedure, the samples did not induce any significant cytotoxic effects on the cells.

To assess the osteogenic capability of MC3T3-E1 cells cultured on the various samples, a series of osteogenic related assays including ALP staining, ALP activity measurements, alizarin red-S staining and its quantified analysis, and real-time quantitative polymerase chain reaction (RT-qPCR) analysis to examine the expression of osteogenic genes were conducted. The ALP staining, illustrated in Fig. 3A, indicated that the micropatterned groups exhibited a subtle purple coloration in contrast to the control group after four days. This differentiation became more evident by the seventh day, as the purple staining intensified in the experimental groups, suggesting elevated ALP activity. This observation aligns with the quantified analysis presented in Fig. 3D, which revealed a markedly enhanced ALP protein activity in the micropattern samples at day 4 relative to the control group. Nevertheless, there was no statistical differences observed between the four micropatterned groups. The ALP activity continued to rise post day 7, with CM60 demonstrating the highest ALP activity, succeeded by CM80, CM40, and CM20. It is important to note that the ALP activity of these four groups remained significantly higher than that of the control group.

The process of mineralization and maturation of osteoblast cells was adeptly visualized through the utilization of alizarin red-S stain, as illustrated in Fig. 3B. It is worth noting that the micropatterned groups exhibited a markedly more pronounced and vivid red coloration relative to the control group at day 14, despite a less prolific presence of calcium nodules. These distinct red hues, in conjunction with calcium nodules, function as vital biomarkers for the progressed phase of osteogenic differentiation, the significance of which was quantitatively assessed Fig. 3E. Nevertheless, by day 21, the micropatterned groups revealed the emergence of calcium nodule deposits, discernibly identifiable through the red nodules. Interestingly, on day 14, both CM40 and CM80 demonstrated a statistically significant increase in mineralization activity when compared to the control group. Furthermore, it is worth mentioning that CM20 and CM60 also exhibited a slightly enhanced capacity for mineralization in comparison to the control group; however, no statistical differences were observed in this regard. In contrast, on day 21, the mineralization activity of the CM20 group experienced a significant rise, reaching levels comparable to that of the CM80 group. It is important to highlight that both groups possess a statistically greater potential for mineralization than that of the control group.

The RT-qPCR analysis further validated the osteogenic differentiation capacity of the MC3T3-E1 cells (Fig. 3F and G). It was observed that the CM80 group exhibited the most pronounced overall gene expressions at both day 7 and day 14, which were significantly higher than the control group (*p* < 0.01). Additionally, on day 7, the CM20 and CM60 groups also displayed significantly higher expression levels of the *Runx2*, *Alp*, *Opg*, and *Ocn* genes. Moreover, the CM40 group exhibited a statistically significant elevation in the expression of the *Runx2* (*p* < 0.01) and *Ocn* (*p* < 0.05) genes, respectively, compared to the control group. However, it is important to note that, apart from the CM80 group, the other three micropatterned groups did not demonstrate any statistically significant difference in the expression of the *Col1* gene. Progressing to day 14, a noteworthy finding was the substantial increase in the expressions of both the *Opg* and *Col1* genes within the CM40 and CM60 groups. Interestingly, the expression levels of the various genes in the CM20 group were either lower or comparable to those in the control group. This intriguing phenomenon could potentially be attributed to the maturation process of the osteoblast cells, as day 14 is considered to be in the late stage of osteogenic differentiation.

#### 3.2.4 Transcriptomic evaluation of osteogenic activity

**Fig. 4:**
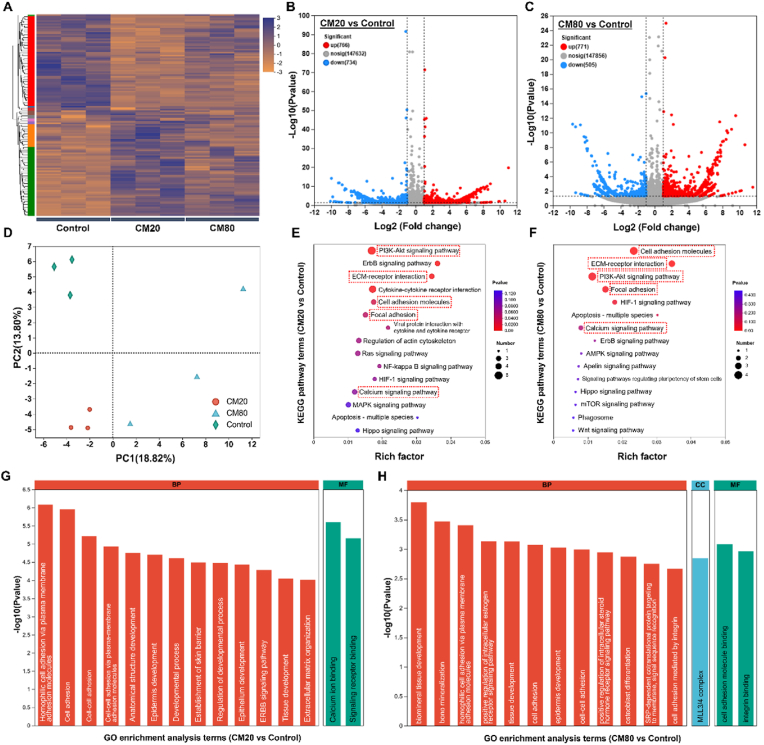
**Transcriptomic analyses of MC3T3-E1 pre-osteoblast cells on the different osteon-inspired concentric micropatterns. A)** Differential gene expression heat-map of MC3T3-E1 cells when cultured onto the different micropatterns; **B & C)** Volcano plots for the DEGs of CM20 vs control, and CM80 vs control, respectively; **D)** PCA representing the variation in gene expressions of MC3T3-E1 cells on the different samples; **E & F)** KEGG enrichment analysis of signalling pathways of CM20 vs Control and CM80 vs Control (Red box indicates upregulated pathways associated with osteogenesis); **G & H)** Gene Ontology (GO) enrichment analysis of the DEGs (BP: Biological Processes, CC: Cellular Components, and MF: Molecular Functions).

The RNA-seq analysis conducted on MC3T3-E1 cells cultivated on osteon-inspired micropatterns elucidated the processes of osteo-differentiation and osteogenesis. The control, CM20, and CM80 groups were examined. The heatmap illustrated in Fig. 4A elucidated pronounced disparities in gene expression patterns among these groups, thereby demonstrating the influence of microgroove width on the osteoblast transcriptome. The principal component analysis (PCA) delineated distinct separations among the experimental cohorts (CM20 represented by red circles, CM80 by blue triangles, and control by green diamonds) as depicted in Fig. 4D. The first principal component (PC1) accounted for 18.82 % of variability, while the second principal component (PC2) explained 13.80 %. An obvious separation was observed between the control group and the CM20 and CM80 groups, indicating that the treatments significantly influenced cellular phenotypic traits. To further explore gene expression differences among the control, CM20, and CM80 groups, a threshold of │log2 fold change (FC)│ >1 and *p* < 0.05 was used to identify significantly expressed genes. The volcano plots presented in Fig. 4 B and C indicated that the CM20 group exhibited 768 upregulated and 734 downregulated genes in comparison to the control, whereas the CM80 cohort demonstrated 771 upregulated and 505 downregulated genes. These findings suggest substantial alterations in gene expression, implying that the CM80 cohort may have activated a greater number of biological pathways.

The KEGG pathway enrichment analysis illustrated in Fig. 4 E and F elucidates the distinct regulatory effects of CM20 and CM80 topological structures on metabolic and signalling pathways associated with implant surfaces. Both micropattern groups showed significant enrichment in the ‘PI3K-Akt signaling pathway’, vital for osteoblast differentiation through its regulation of proliferation, differentiation markers, and mineralization, highlighting its key role in bone formation. Additionally, the ‘focal adhesion’ pathway was enriched in both groups, linking the cell cytoskeleton to the extracellular matrix (ECM) and regulating cellular behavior, including mechanical properties, signaling, and migration. The enrichment of ‘ECM-receptor interaction’ and ‘cell adhesion molecule’ pathways also indicate the biomimetic surface's effectiveness in facilitating essential signaling for osseointegration (consistent with results in Fig. 2, Fig. 3). Collectively, these findings highlight the capacity of modified implant surfaces to affect cellular functions and pathways that are vital for the maintenance and regeneration of bone tissues.

To elucidate the impact of topological modifications in CM20 and CM80 on gene functionality and biological pathways, we performed a GO enrichment analysis (Fig. 4G and H). This analysis evaluated alterations in gene expression within the CM20 and CM80 experimental groups in comparison to the control group, uncovering significant variations in biological processes that are pivotal to bone biology. In the CM20 group, pathways associated to ‘cell adhesion’ (GO ID: 0007155) and ‘ECM organization’ (GO ID: 0030198) were distinctly enriched, supporting the findings in Fig. 2E and F. These pathways are essential for the initial attachment of osteoblastic cells to the implant surface and ensuing deposition of bone matrix. Furthermore, the enrichment of ‘signaling receptor binding’ (GO ID: 0005102) and ‘calcium ion binding’ (GO ID: 0005509) suggests that cell signaling mechanisms play a role in the osteogenic process induced by the implant, particularly due to calcium's essential function in osteoblast mineralization. In the CM80 group, the ‘cell adhesion’ pathway was also enriched, alongside multiple other pathways, including ‘biomineral tissue development’ (GO ID: 0031214), ‘bone mineralization’ (GO ID: 0030282), and ‘osteoblast differentiation’ (GO ID: 0001649), further substantiating the role of surface modifications in promoting osteogenesis. In summary, both CM20 and CM80 enhance osteogenic efficacy by facilitating cell adhesion and activating signaling pathways associated with osteogenesis.

### *3.3* *In vitro* osteoclastic activity

#### 3.3.1 Effect of the osteon-inspired concentric micropatterns on the morphology and distribution of RAW264.7 cells

**Fig. 5:**
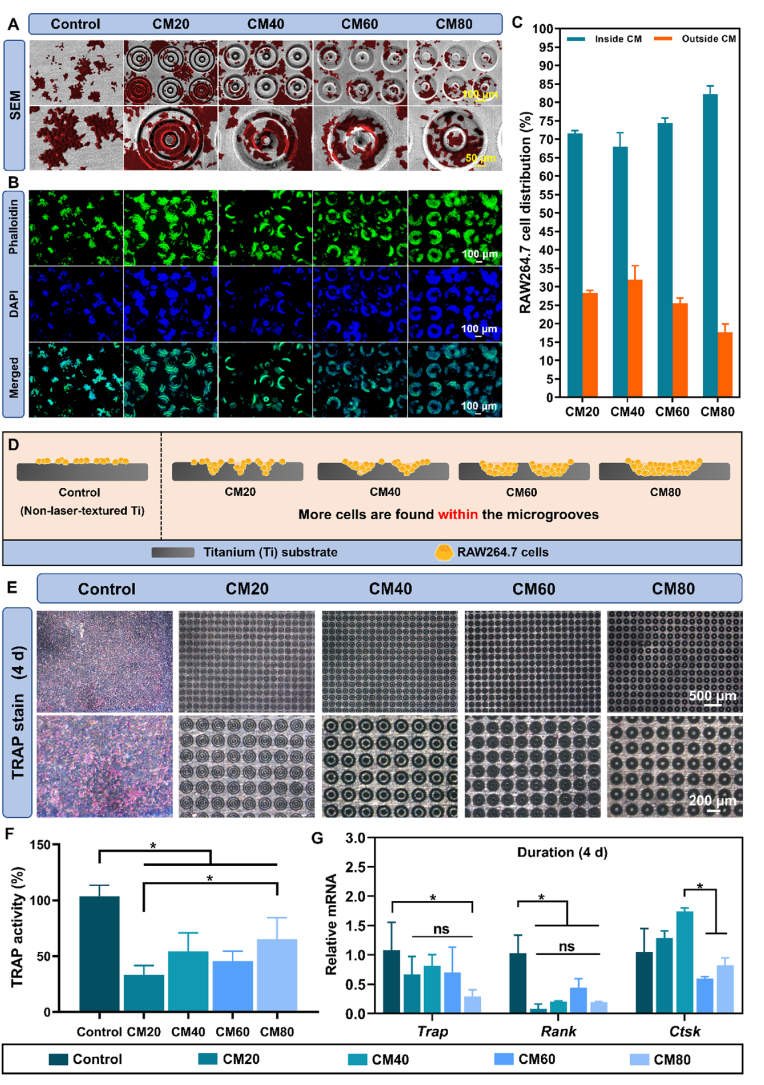
**RAW264.7 macrophage cells behavioural characterization and osteoclastic differentiation and activity on the different osteon-inspired concentric micropatterns. A)** SEM images of RAW264.7 cells on the different samples; **B)** Fluorescence staining of RAW264.7 cells using FITC-phalloidin and DAPI staining on the different samples; **C)** Average distribution of RAW264.7 cells on the samples in percentage; **D)** Schematic diagram depicting the distribution, preference, and selectivity of RAW264.7 cells of the different osteon-inspired concentric micropatterns; **E)** TRAP staining of the different samples after 4 days using hematoxylin dye; **F)** Normalized TRAP activity of the different substrates after 4 d; **G)** Relative gene expressions of osteoclast related genes (*Trap*, *Rank*, *Ctsk*) on the different samples after 4 d ∗*p* < 0.05.

The SEM visualization (Fig. 5A) of RAW264.7 cells on untreated Ti surface showed clustered, sporadic distribution and small, round morphology. Similar morphologies were observed in the micropatterned samples. However, higher magnification images revealed that cells spread out and attached within the micropattern grooves. The formation of pseudopodia was more prominent in these cells. Notably, macrophages exhibited a preference for the wider grooves of the CM80 group, in contrast to osteoblasts, which were less concentrated within the grooves.

The observation was further validated through FITC-phalloidin/DAPI staining (Fig. 5B), which showed cells predominantly occupying the wider grooves of the CM60 and CM80 micropatterns, highlighting the concentricity of the patterns. As groove width narrowed, some cells were found outside the micropatterns. ImageJ software analysis, presented in Fig. 5C, confirmed that cells had a marked preference for the wider grooves of the CM80 group compared to the CM20 group, although they remained primarily within the grooves. This cellular distribution phenomenon is illustrated in Fig. 5D, providing a clear depiction of the spatial arrangement across various micropatterned surfaces.

#### 3.3.2 Osteoclastic differentiation of RAW264.7 cells on the different osteon-inspired concentric micropatterns

The evaluation of the osteoclastic differentiation potential of RAW264.7 cells on the experimental samples involved assessing TRAP activity in relation to the expression levels of the TRAP, RANK, and CTSK genes. To facilitate differentiation into osteoclasts, RANKL and m-CSF were incorporated into the culture medium. TRAP staining images (Fig. 5E) illustrated the most pronounced staining intensity within the control group. Quantitative analysis of TRAP activity (Fig. 5F) revealed that all micropatterned groups exhibited statistically lower TRAP activity in comparison to the control group (*p* < 0.05), with the CM20 group showing the least activity. The RT-qPCR results (Fig. 5G) corroborated these observations, indicating significantly diminished expression levels of the *Trap* and *Rank* genes in the laser-textured micropatterns (*p* < 0.05). Although no statistically significant differences were detected in *Ctsk* expression across the groups, a minor decrease was observed in the CM60 and CM80 groups. Collectively, these results provide compelling evidence that the micropatterns effectively suppress osteoclast differentiation.

### *3.4* *In vitro* fibroblastic activities

#### 3.4.1 Effect of the osteon-inspired concentric micropatterns on the morphology and distribution of HGFs

**Fig. 6:**
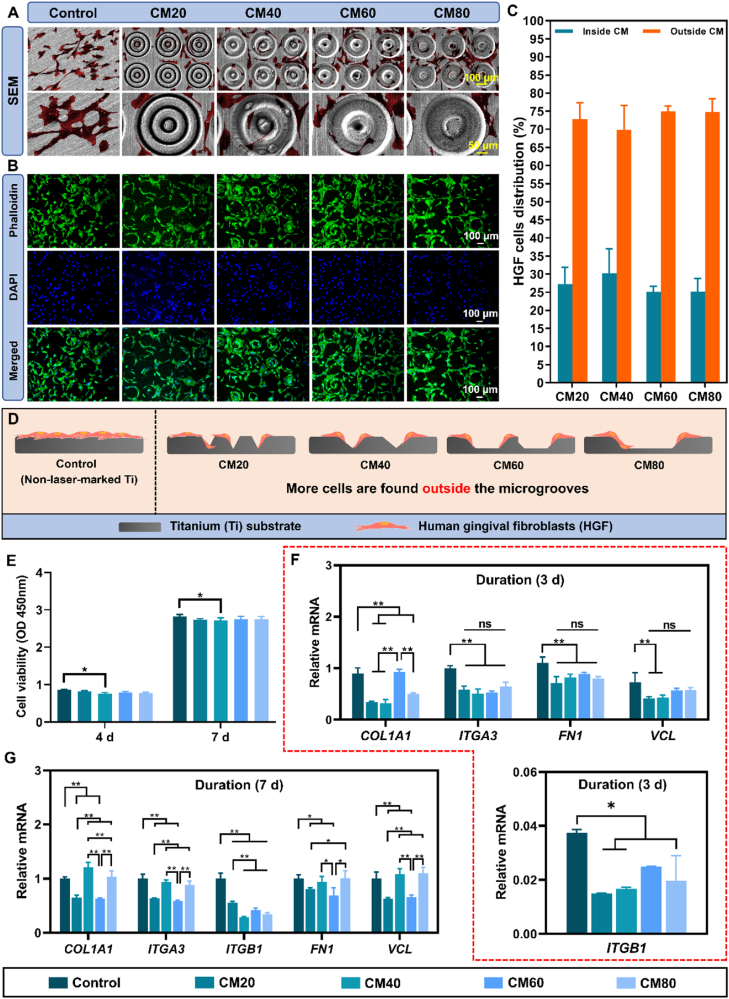
**HGFs behavioural characterization and activity on the different osteon-inspired concentric micropatterns. A)** SEM images of HGFs on the different samples; **B)** Fluorescence staining of HGFs using FITC-phalloidin and DAPI staining on the different samples; **C)** Average distribution of HGFs on the samples in percentage; **D)** Schematic diagram depicting the distribution, preference, and selectivity of HGFs of the different osteon-inspired concentric micropatterns. **E)** CCK-8 of HGFs on the different samples after 4 and 7 days; **F & G)** Relative fibroblastic gene expressions (*COL1A1*, *ITGA3*, *ITGB1*, *FN1*, and *VCL*) measured at 3 and 7 days. ∗*p* < 0.05, ∗∗*p* < 0.01.

The morphology of HGFs was visualized using both SEM and fluorescence microscopy. As shown in Fig. 6A, SEM images reveal that cells in the control group exhibited a random distribution and an elongated shape with filamentous protrusions. In contrast, cells in the micropatterned groups exhibited increased cell area and greater lamellipodia and/or filopodia extension, indicating notable changes in both their spatial distribution and morphology. Notably, the distribution of these cells was markedly different from that of osteoclasts, as described in section 3.3.1 of this study. Specifically, the cells were primarily located on the smooth Ti surface outside the micropatterns and adapted to the edges of the concentric micropatterns. To further validate these findings, double-dye staining images (Fig. 6B) and quantitative analysis (Fig. 6C) confirmed that the cells were indeed positioned outside the micropatterns. This relationship between cell distribution patterns and micropattern design is further illustrated in Fig. 6D.

**Fig. 7:**
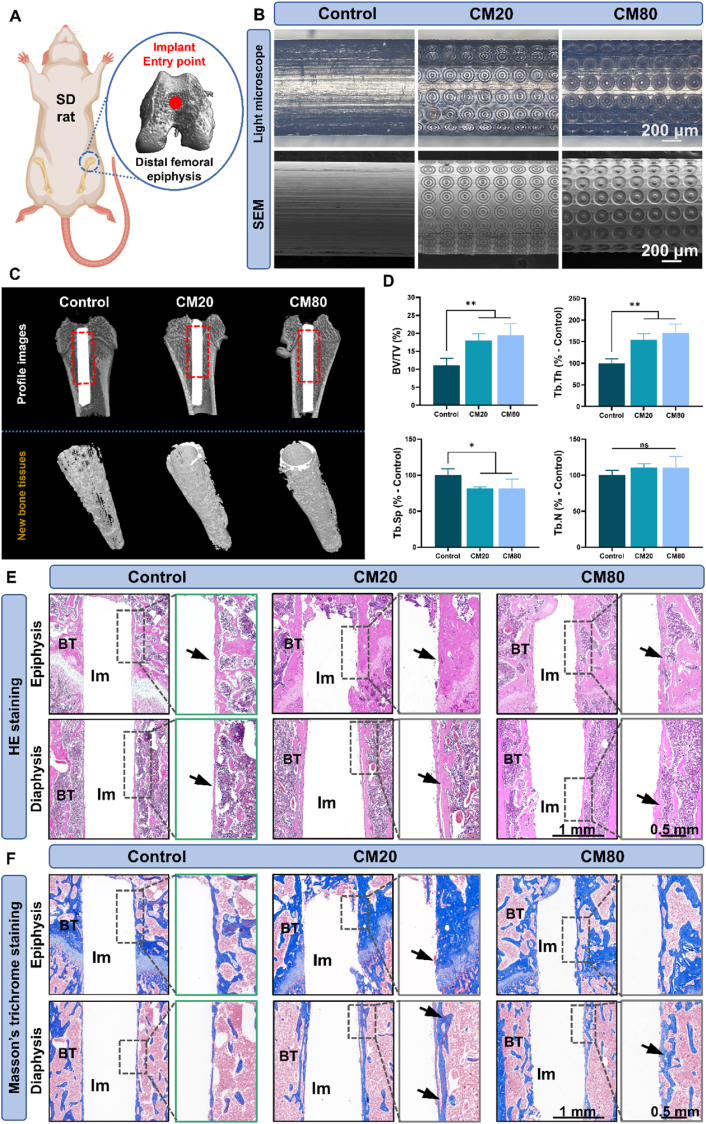
***In vivo* osteogenic bio-characterization of osteon-inspired concentric micropattern-embellished Ti implant samples. A)** Illustration depicting the placement of Ti implants into the bone of SD rats for the *in vivo* study; **B)** Light microscope (top row) and SEM (bottom row) images of the Ti implants and micropatterns used in the *in vivo* investigation (control, CM20, and CM80); **C)** Micro-CT sagittal plane images of the SD-rats’ femurs (top row) and the 3D profile images of the new bone formation around the different implants (bottom row); **D)** Quantified analysis of BV/TV%, Tb.Th, Tb.Sp, and Tb.N; Representative **E)** HE and **F)** MT staining images of bone tissues (BT) surrounding the implants (Im) in the epiphysis and diaphysis regions. New bone formation is indicated with black arrows in the higher magnification of the HE staining images. The blue and red color in the MT staining images represent the low and high maturity of bone tissue, respectively. ∗*p* < 0.05, ∗∗*p* < 0.01.

#### 3.4.2 Cytocompatibility and differentiation of HGFs on the different osteon-inspired concentric micropatterns

As shown in Fig. 6E, no significant cytotoxicity was observed, similar to the viability results of osteoblasts; however, a slight statistical difference was noted between the control and CM40 groups. As illustrated in Fig. 6F, gene expression levels in the micropatterned groups were significantly lower than in the control group, except for *COL1A1*, where the CM60 group showed expression levels comparable to the control. At day 7, a similar trend was observed, particularly in the CM20 and CM60 groups, which exhibited lower expression levels across all genes (Fig. 6G). Conversely, *COL1A1*, *ITGA3*, *FN1*, and *VCL* in the CM40 and CM80 groups showed significant increases, although these were similar to the control group. These results, together with the CCK-8 assay (Fig. 6E) showing comparable viability across all groups, indicate that the micropatterned surfaces modulate fibroblast adhesion and ECM-related gene activity without impairing fibroblast cytocompatibility or overall function.

### *3.5* *In vivo* osteogenic property

#### 3.5.1 Sample characterization and micro-CT evaluation

Three groups (Control, CM20, and CM80) were selected for the surgical implantation procedure on the SD-rats, as illustrated in Fig. 7A. The light microscopy and SEM images in Fig. 7B confirm the successful texturing of the micropatterns onto the Ti implants. Fig. 7C presents the 3D profile images of the bone specimens (top row) alongside the newly generated bone tissue (bottom row) surrounding the implants. Both micropatterned specimens displayed significantly augmented new bone formation compared to the control group. This observation was further verified using the CTan bone volume assessment software. As shown in Fig. 7D, the bone volume fraction (BV/TV%) and trabecular thickness (Tb.Th) for the CM20 (BV/TV%: 17.957 ± 1.94 %; Tb.Th: 161.755 ± 8.02 %) and CM80 (BV/TV%: 19.514 ± 3.24 %; Tb.Th: 176.785 ± 19.14 %) specimens were significantly higher than those of the control group (BV/TV%: 11.154 ± 1.91 %; Tb.Th: 100.0 ± 10.34 %). Additionally, both micropatterned samples demonstrated significantly reduced trabecular separation (Tb.Sp) (CM20: 80.943 ± 1.67 %; CM80: 81.479 ± 13.12 %). However, there were no statistically significant differences in trabecular number (Tb.N) between the micropatterned samples and the control group. It is important to note that, despite lacking statistical significance, the CM80 group demonstrated superior BV/TV% and Tb.Th metrics when compared to the CM20 group. Structure Model Index (SMI) was also calculated and is provided in Table S4. The lower SMI values observed in the CM20 and CM80 groups indicate a more plate-like trabecular morphology relative to the control, which is generally associated with stronger, more mature, and structurally favourable peri-implant bone.

#### 3.5.2 Histological analyses

Both HE and MT staining techniques were utilized to verify the existence of newly generated bone surrounding the implants located in the epiphyseal and diaphyseal regions. The HE staining images depicted in Fig. 7E demonstrate a significantly increased thickness of bone formation (denoted by black arrows) in both the CM20 and CM80 groups relative to the control group across both regions. A comparable pattern is evident in the MT images presented in Fig. 7F, where the CM20 and CM80 groups display a more substantial formation of new bone (indicated by blue coloration) encircling the implants, while the control group reveals a thinner and more irregular distribution of the blue hue that does not consistently encompass the implant. Moreover, the shift from blue to red in the MT staining indicates the maturation process of bone tissues. Notably, red pigmentation within the newly formed bone could be observed (indicated by black arrows) and is more pronounced in the CM20 group.

### 3.6 Potential mechanisms of cell distribution, selectivity, and migration on micropatterns

Recent research indicates that substrate concentricity enhances osteoblast alignment and mechanical properties. Specifically, it has been ascertained that this concentricity not only promotes cellular alignment but also increases the mechanical strength of osteoblasts, reflected in a higher Young's modulus [47]. It was also observed that cell adhesion and spreading can be augmented by the grooves, with cell area and width proportional to groove width [48].

Cell migration is also known to depend on an appropriate balance between adhesion and traction. Insufficient adhesion cannot support stable protrusion, whereas excessive adhesion may impede movement. Although adhesion force was not directly quantified in this study, the CM20 and CM80 groups which demonstrated the most initial attachment of MC3T3-E1 cells, also exhibited the longest migration distances. This correlation is consistent with established principles describing the adhesion–migration relationship, though further work would be required to directly confirm this mechanism [49,50].

One plausible explanation for the phenomenon observed in this study might be that the grooves were overly wide, causing cells to select facile anchorage pathways. This may have enhanced attachment and migration, particularly in the CM20 and CM80 groups (Fig. 2 G, H, and I), where MC3T3-E1 cells adhered to the rough grooves and ridges produced by laser texturing, which osteoblasts prefer. In the CM20 group, cells conformed to narrower grooves before moving onto the ridges, while those in CM80 migrated along the smooth surface while adapting to the outermost ridges of the micropatterns. In contrast, the other groups likely had groove dimensions that were either too narrow for optimal spreading or insufficiently wide to be disregarded. Together, these factors support the interpretation that the micropatterns promote MC3T3-E1 migration.

For macrophage cells, we discerned their predominance within the microgrooves owing to their smaller size. These cells showed a slight inclination for the wider grooves in the CM60 and CM80 groups, as indicated by fluorescent imaging (Fig. 5). The RAW264.7 macrophages exhibited a round, bead-like morphology across all groups, but those in the narrower grooves of the CM20 and CM40 groups also attached to the ridges, suggesting altered spreading. Surface topography can influence cell morphology, affecting subsequent processes such as macrophage polarization, mediated by cytoskeletal tension and RhoA signalling [51,52]. Conversely, fibroblasts were primarily located on the smooth Ti surface rather than in the microgrooves (Fig. 6). This was in line with a previously reported study stating that fibroblast prefers to attach to smooth surfaces [53]. However, some fibroblasts exhibited extensions that attached to the ridges of the micropatterns.

### 3.7 The effects of micropatterns on cellular differentiation and gene expression

We conducted a series of investigations to assess the osteogenic, osteoclastic, and fibroblastic capabilities of the cells on the micropatterns. As depicted in Fig. 3, although cellular proliferation was moderate, the micropatterns significantly augmented the osteogenic potential, as evidenced by elevated levels of ALP, increased mineralization activity, and enhanced expression of osteogenic-related genes. The process of bone formation involves the expression of various complex regulatory genes, such as *Runx2*, *Alp*, *Opg*, *Ocn*, and *Col1*, which were significantly expressed in all micropattern groups, particularly in the CM80 group. In contrast, the osteoclast activity assays (Fig. 5) showed that in the micropattern group, TRAP activity, staining, as well as the genes *Trap*, *Rank*, and *Ctsk* were significantly downregulated. These findings indicates that the micropatterns can effectively induce the differentiation and maturation of osteoblasts while also inhibiting osteoclast differentiation by disrupting the OPG/RANKL/RANK system which plays a significant role in the pathological progression of bone tissue [[54], [55], [56]]. This is consistent with previously reported studies, which also demonstrated the inhibitory effects of concentric micropatterns on osteoclasts [19]. In addition, the upregulation of many biological pathways and metabolic processes related to osteoblast differentiation, growth, and behaviour further validates the osteogenic capacity of these micropatterns, as described in section 3.2.4 and Fig. 4 of this study.

The *in vitro* data regarding osteoblastic and osteoclastic cells were further substantiated by the *in vivo* assessments. Although the animal study does not explicitly evaluate the reduction in osteoclastic bone resorption, it does demonstrate that the micropatterned Ti implants exhibited greater osteoconductivity compared to the control group, as evidenced by significantly increased BV/TV% and Tb.Th levels (Fig. 7D). These results are consistent with *in vitro* analyses (Fig. 3), where the micropatterned samples, particularly in the CM20 and CM80 groups, displayed higher early ALP protein content, mineralization activity, and osteogenesis-related gene expression. *In vivo* histological analyses also confirmed the enhanced osteoconductivity of the micropatterned samples, with HE and MT staining (Fig. 7E and F) showing increased new bone formation. Notably, the MT staining in the experimental groups indicated red pigmentation within the newly formed bone tissue—especially in the CM20 group—suggesting a maturation of the bone not observed in the control group.

Fibroblast activity is critical for the growth and differentiation of fibroblasts, which promote the integration and long-term stability of implants [57]. However, it is also crucial to prevent fibroblast overgrowth, as this can lead to scar tissue formation around implants, which is detrimental to their longevity and may disrupt the attachment of essential cells for bone formation [13]. As shown in Fig. 6, the CCK-8 assay revealed no fibroblast proliferation, and RT-qPCR analysis indicated downregulation of fibroblast-related genes in all micropatterned groups. These findings suggest that micropatterns may effectively modulate fibroblast activity to mitigate overgrowth.

### 3.8 Limitations and future perspectives

Building on the encouraging results of this study, several aspects remain worth exploring to further support the translational development of these osteon-inspired concentric micropatterns. Although the present findings demonstrate strong potential for improving bone–implant integration, additional work will help broaden their applicability and long-term clinical readiness.

In summary, our results indicate that direct PSL-UV laser texturing of osteon-inspired concentric micropatterns effectively modulates cellular behaviors, including distribution, morphology, and gene-level mechanisms, as illustrated in Scheme 1. Based on the findings from this study, we preliminarily conclude that the wider microgroove width of the CM80 group exhibited the highest osteoconductivity, followed by the CM20 group. Notably, these micropatterns demonstrate significantly enhanced osseointegration potential compared to the control group. Herein, osseointegration of Ti implants can be achieved through: 1) modulation of cell contact guidance using micropatterns; 2) upregulation of osteogenic markers; and 3) downregulation of osteoclastic and fibroblastic markers.

While these findings highlight the strong potential of osteon-inspired concentric micropatterns for improving bone–implant integration, several areas offer opportunities for further development. Future studies may assess the performance of these surfaces under clinically relevant compromised conditions such as osteoporosis or diabetes to better understand their translational applicability [58]. As the current work focuses on topographical regulation alone, incorporating bioactive or antibacterial coatings, growth factors, or drug-eluting systems could further enhance early osseointegration and reduce infection risk. Evaluations against widely used clinical implant surfaces (e.g., SLA or acid-etched Ti) and long-term mechanical stability and *in vivo* studies with extended implantation periods will be important next steps. Furthermore, testing in larger animal models may provide a more accurate representation of human bone physiology and better support translational assessment. Together, these future directions represent natural extensions of the present work and may help unlock the full therapeutic potential of osteon-inspired micropatterned Ti implants.

## 4 Conclusion

This study highlights the promising role of biomimetic osteon-inspired concentric micropatterns in enhancing osseointegration. Using PSL-UV laser technology, the precise fabrication of surface features tailored for specific cellular interactions successfully demonstrated enhanced osteoblast activity, regulated and/or inhibited osteoclast and fibroblast activity. The findings provide evidence that varying groove widths influence cellular behavior and bone regeneration, revealing optimal design parameters for improving implant integration. Furthermore, *in vivo* results validated the efficacy of these micropatterns in promoting new bone formation. This research offers a clinically relevant and scalable approach to advancing implant technology, with significant implications for personalized medicine and long-term patient outcomes.

## CRediT authorship contribution statement

**Kendrick Hii Ru Yie:** Writing – original draft, Validation, Methodology, Investigation, Formal analysis, Conceptualization. **Yingyue Sun:** Methodology, Investigation. **Xinhua Gu:** Resources, Methodology, Investigation. **Rui Chen:** Methodology, Investigation. **Zhucheng Liu:** Methodology, Investigation. **Qihong Zhang:** Investigation. **Lifeng Xiong:** Visualization. **Bilal A. Al-Shaaobi:** Investigation. **Ahmed S. Mahany:** Methodology. **Mingliang Yu:** Resources. **Zhennan Deng:** Resources. **Jinsong Liu:** Writing – review & editing, Supervision, Resources, Funding acquisition. **Peng Gao:** Writing – review & editing, Supervision, Software. **Lei Lu:** Writing – review & editing, Writing – original draft, Supervision. **Lihua Xu:** Supervision, Resources.

## Declaration of competing interest

The authors declare that they have no known competing financial interests or personal relationships that could have appeared to influence the work reported in this paper.

## Data Availability

Data will be made available on request.
