# Supplementary material for: Picosecond laser-engineered osteon-inspired concentric micropatterns on titanium implants regulate cellular behaviour to facilitate osseointegration
Source: Mater Today Bio. 2025 Dec 18;36:102704. doi: 10.1016/j.mtbio.2025.102704 (PMC12813317; doi:10.1016/j.mtbio.2025.102704)
Supplement: Multimedia component 1 [file mmc1.docx]

**Supporting information**

**Picosecond Laser-Engineered Osteon-Inspired Concentric Micropatterns on Titanium Implants Regulate Cellular Behaviour to Facilitate Osseointegration**

**Kendrick Hii Ru Yie^1, †^, Yingyue Sun^1, †^, Xinhua Gu^2^, Rui Chen^2^, Zhucheng Liu^2^, Qihong Zhang^1^, Lifeng Xiong^1^, Bilal A. Al-Shaaobi^1^, Ahmed S. Mahany^1^****,** Mingliang Yu^3^, Zhennan Deng^1^, Jinsong Liu^1, *^**,** Peng Gao^1, *^, Lei Lu^1, *^, Lihua Xu^4, *^

1. *School and Hospital of Stomatology, Wenzhou Medical University, Wenzhou, 325027, Zhejiang, China.*
2. *Zhejiang Glister Photonics, Ltd., Zhuji 311800, China*
3. *Zhejiang Trusyou Medical Instruments Co., Ltd.,325000, China*
4. *Department of General Medicine, First Affiliated Hospital, Wenzhou Medical University, Wenzhou 325000, China*

†. These authors contributed equally in this work.

***** **Corresponding authors:**

Lihua Xu, Email address: lihuaxu@wmu.edu.cn

Lei Lu, Email address: [llu2@foxmail.com](mailto:llu2@foxmail.com)

Peng Gao, Email address: penggaocake@foxmail.com

Jinsong Liu, Email address: jinsong0719@wmu.edu.cn

**Table S1.** The parameters used in the design on the osteon-like concentric micropatterns and the fixed laser parameters.

| Groups | CM diameter  (μm) | CM-to-CM spacing  (μm) | Groove depth  (μm) | Groove wall width  (μm) | Groove Width  (μm) | Laser power  (%) | Laser frequency  (kHz) | Scan speed  (mm/s) |
| --- | --- | --- | --- | --- | --- | --- | --- | --- |
| CM20 | 200 | 30 | 20 | 15 | 20 | 30-35 | 300 | 200 |
| CM40 | 200 | 30 | 20 | 15 | 40 | 30-35 | 300 | 200 |
| CM60 | 200 | 30 | 20 | 15 | 60 | 30-35 | 300 | 200 |
| CM80 | 200 | 30 | 20 | 15 | 80 | 30-35 | 300 | 200 |

**Table S2.** The primers used for RT-qPCR in this study to assess osteogenesis, osteoclastogenesis, and fibrogenesis.

| Target genes | Origin | Primer sequence |
| --- | --- | --- |
| *Alp* | Mouse | F: 5’-AGCGACACGGACAAGAAGC-3’  R: 5’-GGCAAAGACCGCCACATC-3’ |
| *Col1* | Mouse | F: 5’-GATGTTGAACTTGTTGCTGAGGG-3’  R: 5’-GGCAGGCGAGATGGCTTATT-3’ |
| *Opg* | Mouse | F: 5’-GCCCAGACGAGATTGAGAG-3’  R: 5’-CAGACTGTGGGTGACGGTT-3’ |
| *Ocn (Bglap)* | Mouse | F: 5’-AGATTGTTGGGGCACAAG- 3’  R: 5’-CCTTCAGCAGGGAAACCG- 3’ |
| *Runx2* | Mouse | F: 5’-GCCGTAGAGAGCAGGGAAGA-3’  R: 5’-CTGGCTTGGATTAGGGAGTCA-3’ |
| *Rank* | Mouse | F: 5’-CAGGGATCGATCGGTACAGT-3’  R: 5’-GTTTGAGACCAGGCTGGGTA-3’ |
| *Trap (Acp5)* | Mouse | F: 5’-TACCTGTGTGCACATGACC-3’  R: 5’-CAGATCCATAGTGAAACCGC-3’ |
| *Ctsk* | Mouse | F: 5’-TGTATAACGCCACGGCAAA-3’  R: 5’-GGTTCACATTATCACGGTCACA-3’ |
| *Gapdh* | Mouse | F: 5’-CTCGTCCCGTAGACAAAATGGT-3’ |
|  |  | R: 5’-GAGGTCAATGAAGGGGTCGTT-3’ |
| *COL1A1* | Human | F: 5’-TCTAGACATGTTCAGCTTTGTGGAC-3’  R: 5’-TCTGTACGCAGGTGATTGGTG-3’ |
| *FN1* | Human | F: 5’-ACCTACGGATGACTCGTGCTTTGA-3’  R: 5’-CAAAGCCTAAGCACTGGCACAACA-3’ |
| *ITGB1* | Human | F: 5’-TGTGTCAGACCTGCCTTGGTG-3’  R: 5’-AGGAACATTCCTGTGTGCATGTG-3’ |
| *ITGA3* | Human | F: 5’-ACTATACACTCCAGACCTCCCTT-3’  R: 5’-ACAGTTTTCATGCCACACTCACC-3’ |
| *VCL* | Human | F: 5’-TTGATGGGTCAAGGGGCATC-3’  R: 5’-CACCACCTCTGCCACTGTAA-3’ |
| *GAPDH* | Human | F: 5’-GCACCGTCAAGGCTGAGAAC-3’ |
|  |  | R: 5’-TGGTGAAGACGCCAGTGGA-3’ |

**Table S3.** The elements detected through EDS of the different samples and their corresponding atomic percentage (At %) and weight percentage (Wt %).

| Group | Elements | | | | | |
| --- | --- | --- | --- | --- | --- | --- |
|  | Ti | | O | | C | |
|  | *At %* | *Wt %* | *At %* | *Wt %* | *At %* | *Wt %* |
| Control | 74.90 | 89.98 | 24.53 | 9.85 | 0.57 | 0.17 |
| CM20 | 54.37 | 78.13 | 45.27 | 21.74 | 0.36 | 0.13 |
| CM40 | 56.12 | 79.33 | 43.37 | 20.49 | 0.51 | 0.18 |
| CM60 | 58.91 | 81.13 | 40.69 | 18.73 | 0.40 | 0.14 |
| CM80 | 57.60 | 80.31 | 41.85 | 19.50 | 0.55 | 0.19 |

**Table S4.** Structure Model Index (SMI) values calculated from micro-CT analysis of peri-implant trabecular bone.

| Group | Structure Model Index (SMI; mean ± SD) |
| --- | --- |
| Control | 1.13 ± 0.03 |
| CM20 | 0.63 ± 0.15 |
| CM80 | 0.65 ± 0.17 |

**
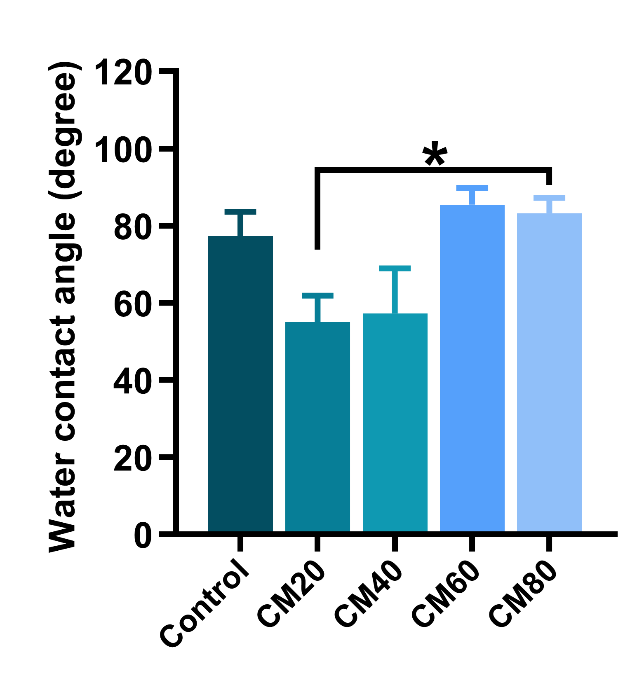
**

**Fig. S1.** WCA of the different samples. *p < 0.05.


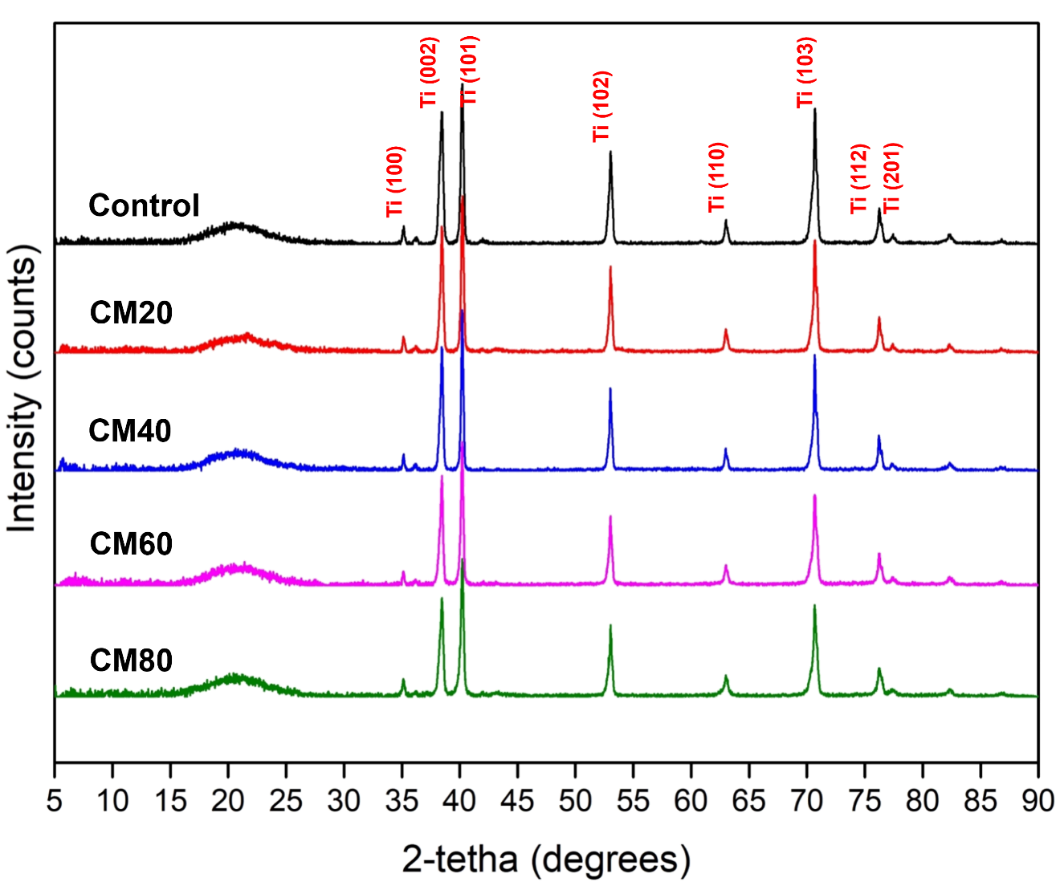


**Fig. S2.** XRD readings of the different samples.

**
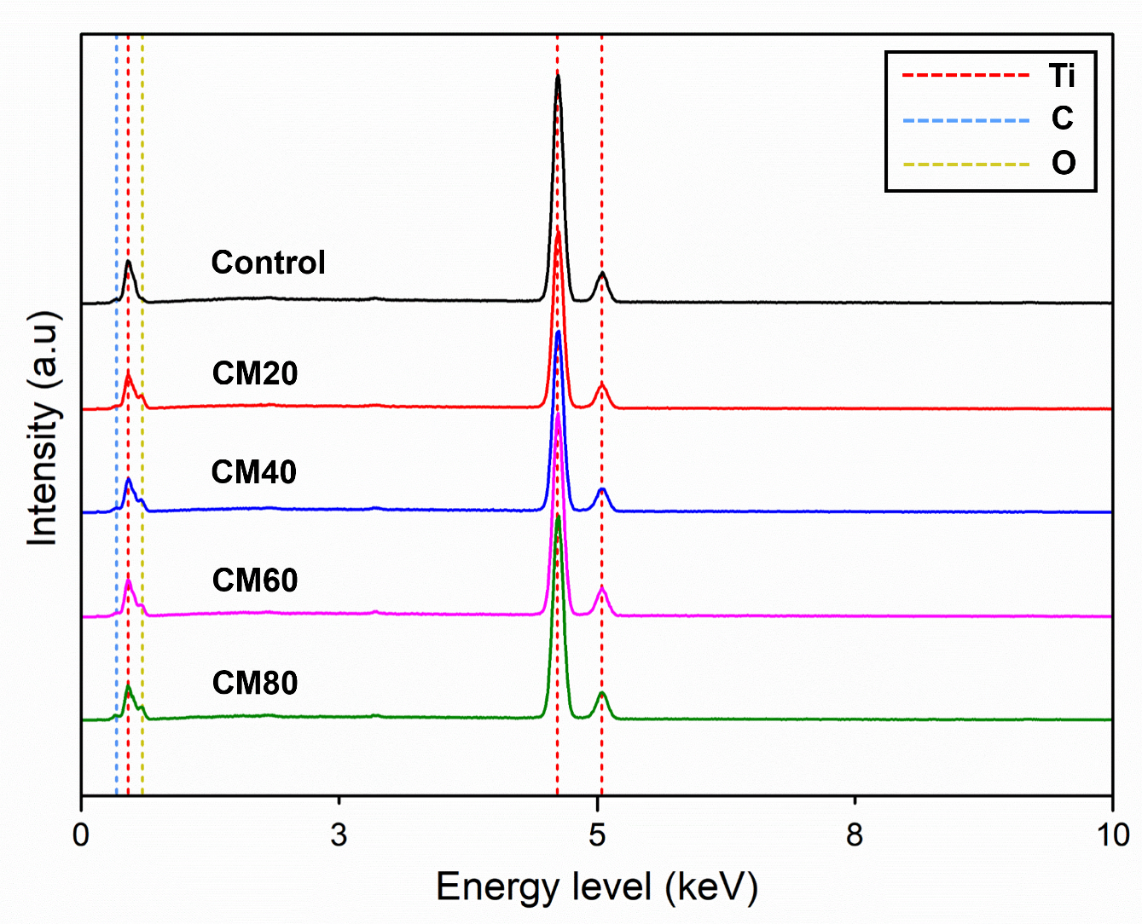
**

**Fig. S3.** EDS analysis of the different samples.
